# Immediate impact of extremity manipulation on dual task performance: a randomized, crossover clinical trial

**DOI:** 10.1186/s12998-021-00366-5

**Published:** 2021-02-05

**Authors:** Christopher A. Malaya, Joshua Haworth, Katherine A. Pohlman, Dean L. Smith

**Affiliations:** 1 Center for Neuromotor and Biomechanics Research, University of Houston, Houston, TX USA; 2 Research Center, Parker University, Dallas, TX USA; 3 Department of Human Movement Science, Oakland University, Rochester, MI USA; 4 Department of Kinesiology and Health, Miami University, Oxford, Ohio USA; 5 Essence of Wellness Chiropractic Center, Eaton, OH USA

**Keywords:** Chiropractic manipulation, Extremity, Coordination, Motor control, Postural balance

## Abstract

**Background:**

Previous research demonstrated that manipulation of the extremities was associated with changes in multisegmental postural sway as well as improvement in a lower extremity balancing task. We were interested if these effects would extend to an upper extremity task. Our aim in this study was to investigate whether extremity manipulation could influence dual task performance where the explicit suprapostural task was balancing a water filled tube in the frontal plane.

**Methods:**

Participants were healthy volunteers (aged 21–32 years). Upper- or lower-extremity manipulations were delivered in a participant and assessor blinded, randomized crossover, clinical trial. Postural (center of pressure) and suprapostural (tube motion) measurements in the frontal plane were made pre-post manipulation under eyes open and eyes closed conditions using a BTrackS™ force plate and a Shimmer inertial measurement unit, respectively. Pathlength, range, root mean square and sample entropy were calculated to describe each signal during the dual task performance.

**Results:**

There was no main effect of manipulation or vision for the suprapostural task (tube motion). However, follow-up to interaction effects indicates that roll pathlength, range and root means square of tube motion all decreased (improvement) following lower extremity manipulation with eyes open. Regarding the postural task, there was a main effect of manipulation on mediolateral center of pressure such that pathlength reduced with both upper and lower extremity manipulation with larger decreases in pathlength values following upper extremity manipulation.

**Conclusion:**

Our findings show that manipulation of the extremities enhanced stability (e.g. tube stabilization and standing balance) on performance of a dual task. This furthers the argument that site-specific manipulations influence context specific motor behavior/coordination. However, as this study focused only on the immediate effects of extremity manipulation, caution is urged in generalizing these results to longer time frames until more work has been done examining the length of time these effects last.

**Trial registration:**

Clinicaltrials.gov, NCT03877367**,** Registered 15 March 2019. Data collection took place July 2019.

## Background

Maintaining an upright stance requires torques to be generated around the ankles, knees, hips and even the upper extremities [1–4]. Movement of one part of the body entails compensatory adjustments elsewhere for bi-pedal individuals to maintain their center of mass above their base of support and thus remain standing. In both posture and goal directed “suprapostural” activities, such as reaching or balancing an object with the upper extremities, the control of movement depends on the continuous and accurate regulation of many muscles, joints and limbs [5, 6]. It has been suggested that during these dynamic activities, the arms and trunk may be used to generate restorative torques to reduce the angular momentum of the body [1], which would require proprioceptive information relating to the position of not only the limbs, but also the trunk and head. Accordingly, dynamic postural control seems to require whole body coordination.

Evidence for neurologically based mechanisms of action for spinal manipulative therapy include central changes in sensorimotor and cortical integration [7, 8], as well as peripheral changes to volitional elbow flexor activity [9] and joint position sense [10]. While it has been suggested that chiropractors examine posture from a dynamic perspective, including suprapostural behaviors [4], few research studies have been conducted in this area. There are even fewer research studies exploring the effects of extremity joint manipulation on postural dynamics and/or sensorimotor integration.

Human postural control is tantamount to one’s ability to find and maintain bipedal balance in an environment, and the neuromusculoskeletal system is the mediator of and primary responder to corrective movements meant to maintain stability. It is important then, that health research examine topics that will add to our basic understanding of how people’s movements and behaviors, as well as their anatomy and physiology, change with joint manipulations. We previously conducted a study that examined the effect of upper and lower extremity manipulation on posture and balance [11]. We found that lower extremity manipulation influenced several dynamic measures of postural sway while standing on both the ground and rocker board. That is, extremity joint manipulation of the lower extremities improved the organization of sway for the trunk (anterior-posterior direction) and rocker board (medial-lateral direction) and extremity manipulation of the upper extremities reduced roll range and pathlength on the lower extremity-based rocker board task. We postulated that these effects could be due to a change in sensory input and respective motor output leading to behavioral modifications such as restorative torques and postural sway. Furthermore, the magnitude and direction of the sensorimotor change appeared to be responsive to the task being performed and the joint being manipulated.

As a follow-up to that study, we examined the effects of upper and lower extremity joint manipulations on an upper body task, holding a water-filled tube parallel to the ground. Given that holding a tube while standing is a dual task, we assessed participant’s performance with both posturography and an inertial measurement unit (IMU). Our aim was to investigate whether extremity manipulation could influence dual task performance where the explicit suprapostural task was balancing a water filled tube in the frontal plane by the upper extremity. Since vision influences tactile processing [12, 13], we tested participants while standing with both eyes open and closed. We hypothesized: 1) both upper and lower extremity manipulation would reduce tube roll parameters, as well as mediolateral postural sway; 2) upper extremity manipulation would reduce tube roll to a greater extent, and that lower extremity manipulation would reduce postural sway to a greater extent; 3) the presence or absence of vision would also influence task performance.

## Methods

### Participants

A sample of 23 healthy chiropractic students (78% male) between the ages of 21 and 32 (mean age ± standard deviation: 27.4 ± 2.7 years) were recruited. Participants were recruited from the Parker University student body with flyers posted around campus. Interested participants contacted the study coordinator from information listed on the flyer and were scheduled for screening. Screening and testing occurred on the same day. Eligible participants were between the ages of 18 and 35, were not pregnant, had no known musculoskeletal, neurological or visual impairments that could impact their ability to stand upright and were asked to refrain from any chiropractic manipulations outside of the study itself. Written informed consent was obtained from each participant prior to the start of experimental procedures. Approval to conduct this study was granted by the Institutional Review Board at Parker University (#A-00186), in accordance with the Declaration of Helsinki. All testing was performed at Parker University’s Research Center. This study was registered at ClinicalTrials.gov; (NCT number: NCT03877367). Data collection took place July 2019.

The within subjects sample size for this study was based on a power analysis conducted by an independent biostatistician. The sample size for this study was based on pre-post standard deviations (SDs) of mean changes in mediolateral (ML) rocker board sample entropy (SampEn) from our previous study [11]. As such, twenty participants per group would provide at least 80% power to detect a medium to large effect size of 0.335 at a 0.05 level of significance.

### Study design

**Fig. 1:**
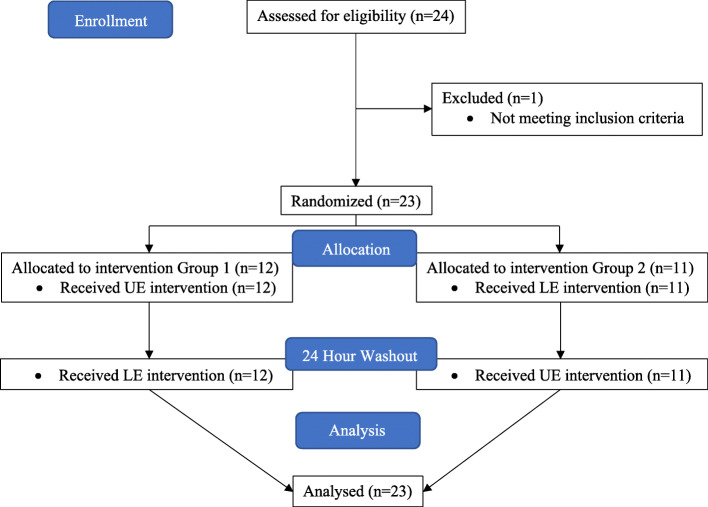
CONSORT

This was a crossover trial. We used a within-subjects study design to determine the influence of each of the conditions on our dependent variables and to control for the potential influence of individual differences. The study chiropractor and data collectors were blinded. The study chiropractor was aware of treatment assignment but was blinded to the values of the measurements taken. Data collectors were aware of measurement values but were blinded to treatment assignment. Participants were enrolled by the study coordinator after which they were block randomized into two different groups by a custom MATLAB script. The script was run and results delivered to the doctor performing the interventions by a graduate student not involved in data collection. Group one received an upper extremity manipulation series on the first day and, after a 24-h washout period, returned and received a lower extremity manipulation series. Group two received a lower extremity manipulation on the first day and an upper extremity manipulation series on the second day (see Fig. 1). Participants were assessed on dual task performance no more than 2 min before and no more than 2 min after receiving joint manipulations on both days.

The joint manipulations were performed distally to proximally and were distal radioulnar, humeroulnar, and glenohumeral (upper extremity series) and tibiotalar, tibiofemoral, and coxofemoral joints (lower extremity series), respectively. Both series were performed bilaterally for each participant and all manipulations were performed by an experienced chiropractor with greater than 10 years clinical experience.

### Dual task

**Fig. 2:**
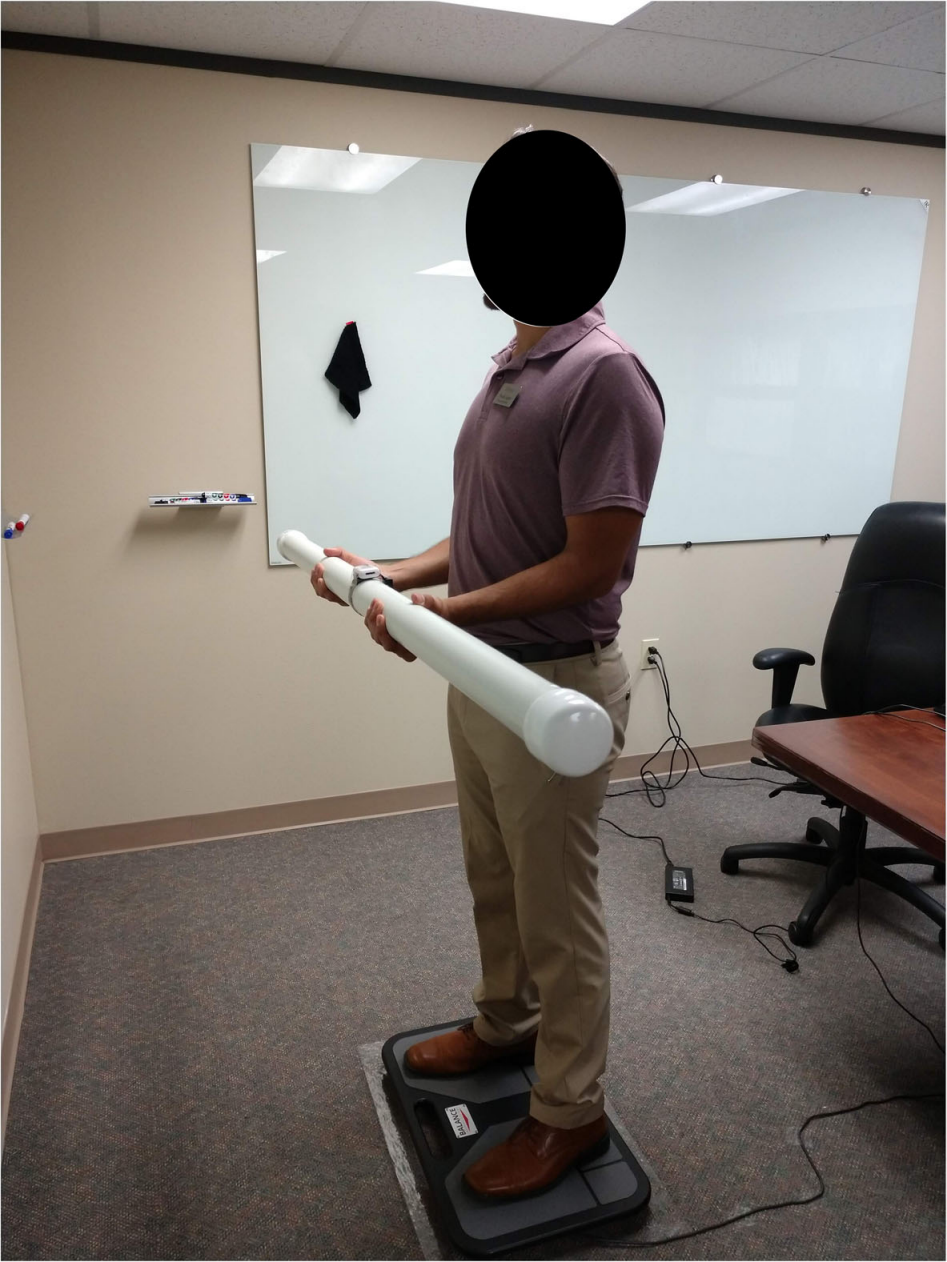
Experimental Setup

Participants were asked to stand comfortably on a Balance Tracking System (BTrackS™, San Diego, CA) force plate with their elbows bent at 90 degrees and hands in line with elbows. They were then handed a capped 2″ diameter PVC tube (60.5″ long, weight: 4.60 lbs) half-filled with water (see Fig. 2). Previous research using a similar water filled tube has found that when lifted, water within the tube moves and immediately demands control, stabilization and greater muscle engagement particularly with paraspinal, deltoid, and abdominal muscles [14]. Participants wore comfortable, athletic shoes during the testing sessions and the same pair of shoes across days. This was to ensure that standing test conditions mirrored normal, everyday standing as closely as possible. Wearing a standard/athletic shoe compared to barefoot does not seem to significantly affect postural balance or range of motion particularly on a firm surface [15–17].

Participants were instructed to “hold the tube level with the ground”. Participants held the tube for 30 s each under the eyes closed and eyes open conditions. Visual condition was randomized by participant using a custom MATLAB script, and this test order was maintained for the duration of the study. Data capture began immediately after the study associate released the tube into the participants hands.

### Data collection

The tube was fitted with an IMU (Shimmer Sensing) that collected kinematic data. Data were streamed wirelessly at 51.2 Hz to the Consenys v.1.5.0 software platform (Shimmer Sensing) and exported for processing.

Center of pressure (COP) data were collected by a BTrackS™ (Balance Tracking Systems) force plate. Data were acquired through the Explore Balance software application (Balance Tracking Systems, version 2.0.4) at 50 Hz.

Data from the tube and force plate were processed by a custom Matlab script (Matlab R2018b:9.5.0.944444). Trial duration for each participant was 30 s with eyes open and 30 s with eyes closed, respectively. All sample data were processed. SampEn, path length (pathlength), range, and root mean square (RMS) were calculated for the roll direction of the tube sensor (in degrees) as well as for the ML COP of the force plate (in centimeters). SampEn is a measure of the complexity or repeatability of a physiological time series [18, 19]. In this study, pathlength is the cumulative distance traveled by the tube sensor or the participant and range is distance between the maximum excursions of the tube or the participant’s mediolateral CoP [19]. RMS is a measure of the magnitude that the tube sensor or the participant’s CoP varies with respect to the mean location [19].

### Data analysis

Change scores (post minus pre) were calculated for SampEn, pathlength, range and RMS for site of manipulation and visual condition. These change scores served as the dependent variable in separate analyses for the IMU and COP measures. All upper and lower extremity manipulation and eyes open/eyes closed change scores were entered into the analysis. Statistical analysis of COP and IMU data was performed using a 2 × 2 within-subjects ANOVA (2 factors: site of manipulation and visual condition, with 2 levels each: upper and lower extremity manipulation, and eyes open and eyes closed, respectively). Post hoc tests were performed using Bonferroni corrected pairwise comparisons. All analyses were conducted using IBM SPSS Statistics for Windows, Version 25.0 (IBM Corp., Armonk, NY, USA). Statistical significance was set at an alpha value of 0.05.

## Results

### Participants

One participant was excluded from randomization due to failure to meet inclusion criteria (outside of age range). No participants were lost to follow-up and all collected data were used in the analysis. There were no adverse events or unintended effects during the course of the study.

### Suprapostural task

There were no main effects of manipulation or vision for any of the measured dependent variables. See Table 1 for mean change scores and standard deviations for tube roll motion. The main effect of manipulation was not significant for roll path length F(1,22) = 0.079, *p* = 0.78; range F(1,22) = 0.31, *p* = 0.59; RMS F(1,22) = 0.03, *p* = 0.87; or SampEn F(1,22) = 0.01, *p* = 0.93. The main effect of vision was not significant for roll path length F(1,22) = 0.84, *p* = 0.37; range F(1,22) = 0.38, *p* = 0.54; RMS F(1,22) = 0.00, *p* = 0.98; or SampEn F(1,22) = 0.18, *p* = 0.68. However, interactions for roll path length, roll range, roll RMS and roll SampEn were all significant. Post hoc pairwise comparisons were performed for significant interactions with a Bonferroni adjustment applied.

**Table 1:** Mean change scores and standard deviations for tube roll motion as measured by IMU

Variable	Lower Extremity		Upper Extremity	
	Eyes Open	Eyes Closed	Eyes Open	Eyes Closed
Roll PathLength	−190.82 (387.22)	17.74 (343.22)	−66.82 (535.88)	− 163.80 (453.50)
Roll Range	−6.01 (10.19)	−1.10 (7.74)	−0.71 (12.30)	−3.61 (8.28)
Roll RMS	−0.60 (0.89)	−0.15 (0.78)	− 0.19 (1.21)	−0.64 (1.19)
Roll SampEn	0.11 (0.16)	0.06 (0.14)	0.04 (0.14)	0.12 (0.20)

Mean (SD) values are listed for each condition. Mean values represent mean change scores between pre-post conditions. Path Length, Range and RMS are measured in degrees (deg). SampEn is a unitless measure.

For roll path length, the significant interaction, F(1,22) = 11.92, *p* = 0.002, ηp^2^ = 0.351 with follow up comparisons showed a significant effect of lower extremity manipulation with vision (*p* = 0.022). Specifically, there was a reduction in roll path length following lower extremity manipulation with eyes open (mean difference = − 190.82 degrees; 95% CI, − 358.27 to − 23.37) compared to eyes closed (mean difference = 17.74 degrees; 95% CI, − 130.69 to 166.16).

For roll range, the significant interaction, F(1,22) = 7.27, *p* = 0.013, ηp^2^ = 0.248 with follow up comparisons showed a significant effect of lower extremity manipulation with vision (*p* = 0.030). Roll range reduced to a greater extent following lower extremity manipulation with eyes open (mean difference = − 6.01 degrees; 95% CI, − 10.42 to − 1.60) compared to eyes closed (mean difference = − 1.10 degrees; 95% CI, − 4.45 to 2.25).

For roll RMS, the significant interaction, F(1,22) = 12.89, *p* = 0.002, ηp^2^ = 0.37 with follow up comparisons also showed a significant effect of lower extremity manipulation with vision. Roll RMS also reduced to a greater extent following lower extremity manipulation with eyes open (mean difference = − 0.60 degrees; 95% CI, − 0.99 to − 0.22) compared to eyes closed (mean difference = − 0.15 degrees; 95% CI, − 0.48 to 0.19).

For roll SampEn, the significant interaction, F(1,22) = 4.95, *p* = 0.037, ηp^2^ = 0.184 did not produce any significant follow up comparisons.

### Postural task

There was a main effect of manipulation for ML pathlength, F(1,22) = 9.92, *p* = 0.005, ηp^2^ = 0.31. Pre-manipulation values were larger than post manipulation values. Both manipulation types reduced ML COP pathlength. Upper extremity manipulation reduced COP to a greater extent (mean difference = − 7.14 cm; 95% CI, − 10.80 to − 3.47) than lower extremity manipulation (mean difference = − 2.83 cm; 95% CI, − 5.22 to − 0.43). No main effect of vision was found (F(1,22) = 0.05, *p* = 0.82), and there was no interaction of vision and manipulation (F(1,22) = 0.15, *p* = 0.70).

There was also a main effect of manipulation for ML RMS, F(1,22) = 5.28, *p* = 0.032, ηp^2^ = 0.19. Mean pre-manipulation values were larger than post manipulation values for lower extremity manipulations, hence lower extremity manipulations reduced COP RMS (mean difference = − 0.25 cm; 95% CI, − 0.52 to 0.01). Mean pre-manipulation values were less than post manipulation values for the upper extremity and thus increased COP RMS (mean difference = 0.29 cm; 95% CI, − 0.054 to 0.62). No main effect of vision was found (F(1,22) = 1.31, *p* = 0.27), and there was no interaction of vision and manipulation either, (F(1,22) = 0.03 *p* = 0.87). See Table 2 for mean change scores and standard deviations for ML force plate measurements.

**Table 2:** Mean change scores and standard deviations for ML force plate measurements

Variable	Lower Extremity		Upper Extremity	
	Eyes Open	Eyes Closed	Eyes Open	Eyes Closed
ML PathLength	−3.35 (6.56)	−2.31 (7.43)	−7.04 (9.79)	− 7.23 (12.01)
ML Range	−0.13 (0.48)	− 0.06 (0.81)	−.31 (0.56)	− 0.45 (1.21)
ML RMS	− 0.30 (0.73)	− 0.21 (0.69)	0.22 (0.75)	0.35 (0.89)
ML SampEn	−0.00 (0.02)	−0.00 (0.04)	− 0.01 (0.04)	−0.01 (0.04)

Mean (SD) values are listed for each condition. Mean values represent mean change scores between pre-post conditions. Path Length, Range and RMS are measured in centimeters (cm). SampEn is a unitless measure

The main effect of manipulation was not significant for range F(1,22) = 3.38, *p* = 0.08 or SampEn F(1,22) = 3.12, *p* = 0.09. The main effect of vision was not significant for range F(1,22) = 0.05, *p* = 0.82 or SampEn F(1,22) = 0.04, *p* = 0.84. No significant main effects or interactions were found for the range (F(1,22) = 0.48, *p* = 0.50), or SampEn (F(1,22) = 0.00, *p* = 0.99) dependent measures involving ML sway on the force plate.

## Discussion

Participants in this study were asked to simultaneously perform a postural and suprapostural dual task immediately before and immediately after receiving either a lower or upper extremity manipulation. Both upper- and lower-extremity manipulation influenced dual task performance as compared to initial testing. Lower extremity manipulation with eyes open significantly reduced tube motion as assessed by roll pathlength, range and RMS, whereas both upper and lower extremity manipulation reduced COP movement on a force plate as assessed by ML postural sway. SampEn, a measure of movement structure and periodicity, provided no further insight into tube roll or postural sway, in contrast to our expectations from previous work.

Research on spinal manipulation has shown changes in volitional muscle activity [9], voluntary range of motion [20], biomechanical and structural changes [21], complex whole-body motor response task [22], movement time [23] and joint position sense [24]. As their effects extend beyond the local anatomical area of manipulation, it has been postulated that these changes may be driven by downstream cortical stimulation rather than spinal or local influences [25]. Similarly, in this study, we found that chiropractic manipulation of the extremities influenced both upper and lower extremity-based task performance.

In this study, participants’ performance on the tube balancing task was modulated by an interaction between lower extremity manipulation and the participants’ visual condition. In the eyes open condition, lower extremity manipulation led to decreased values of tube roll parameters, indicating enhanced stability. The importance of visual information to joint manipulative effects is inherently pragmatic/useful, as most chiropractic patients are utilizing visual information throughout their daily activities; however, it is still not known how the central nervous system combines relevant somatosensory and visual information for such control. One possibility may be that (“noninformative”) vision improves haptic perceptions of peripersonal space [13]. More work is needed to better understand the relationship between manipulation and vision.

The interplay between the visual and somatosensory systems has been elicited in many postural studies, particularly in work concerning muscle and tendon vibration. Mancheva et al. [26] found that motor evoked potentials from transcranial magnetic stimulation during tendon vibration varied depending on whether subjects’ eyes were open or closed [26]. Lackner and Levine [27] showed simultaneous vibration of the neck and Achilles tendons could induce nystagmoid eye movements and Bove et al. [28] found that vibration over postural muscles could alter proprioceptive integration, leading to changes in body tilt and rotation [27, 28]. From our findings, we propose that joint manipulation of the extremities may stimulate the same primary and secondary afferents stimulated by muscular and tendon vibration and that these changes in somatosensation can facilitate cortical changes and alter motor outputs [25, 29–31].

According to Pacheco et al. [32] in the ecological theory of perception and action, enhanced stability (e.g. tube stabilization) occurs from the attunement of the perceptual systems to task dynamics together with modifications of action as task and intrinsic dynamics cooperate and/or compete. Chiropractic manipulation may then modulate the properties of the perceptual-motor workspace of participants. The prevailing thought on the neurophysiological impact of spinal/extremity manipulation is one of perceptual attunement brought about by mechanisms related to greater afferentation by peripheral receptors [33–35]; however, our consistent interaction effects suggest the modulation of visual perception may also be a possibility. Furthermore, the action capabilities of the participant are likely promoted by enhanced neural drive through supraspinal, spinal or extremity-based mechanisms [25, 36, 37].

As described in the introduction, previous work by this team found that ipsilateral upper and lower extremity manipulations affected participant performance during a lower extremity balance task (standing on a rocker board) [11]. In that study, both upper and lower extremity manipulations led to decreased pathlength as measured on a rocker board. While participants in the current study stood on a force plate (rather than a rocker board), again, both upper and lower extremity manipulation led to decreased ML pathlength (in this case COP pathlength). This is particularly interesting as this effect is found irrespective of whether the manipulations involved a single limb (previous study) or both limbs (current study). While comparing the magnitudes of single vs bilateral limb manipulation effects would be overly speculative given the differences between the two studies, this is an interesting question that could be addressed in future studies.

It is important to note that we did not capture the segmental (or multi-segmental) strategies used by participants in this study. Collecting such data may be able to resolve why contrary to our hypothesis, upper extremity manipulation had no effect on tube stabilization, but did reduce ML postural sway. Such information would also likely explain why lower extremity manipulation consistently aided tube stability. Despite the opacity of strategies utilized, participant performance is still consistent with an ecological model; joint manipulation afforded participants greater stability during dual task performance. We suggest that further research is necessary to understand how extremity manipulations afforded the aforementioned improvement in performance. Nonetheless, we feel these results are exciting as they represent some of the initial steps in understanding a non-invasive means of potentially altering an individual’s sensory integrative state. As manual therapy, including extremity manipulation, may have a role to play in improving postural stability [38], we feel this work could lead to new and beneficial therapies aimed at preserving movement and coordination, and mitigating falls and fall risk in affected populations.

While these results are novel, they require replication. This study is also limited in that it examined only healthy, asymptomatic, adult participants. While many interesting effects can become more pronounced in clinical populations, many effects can also disappear entirely. These results do not currently, and may not necessarily generalize beyond a healthy, asymptomatic population. Future work should investigate these effects in special populations, and, particularly, the elderly, where balance and falls are major factors in injury and loss of independence. We also did not examine the effects of chiropractic manipulation beyond an immediate (less than 2 min) time frame. Future research should investigate the temporal effects of extremity manipulation on dual task performance beyond an immediate time frame. It is also possible that manipulation applied to different joints can elicit individual responses of potentially differing magnitudes. In this study, the order of manipulation (by series) was also kept constant. Future work should investigate any potential differences in the order and location of selected manipulations.

## Conclusion

Joint manipulations of the upper and lower extremities enhanced stability across a postural / suprapostural dual task in the presence of visual information. Extremity manipulations also appear to influence motor behavior beyond the local anatomical area of the joint being manipulated. These results suggest that a centrally integrative mechanism – similar to that of spinal manipulation – is also present with manipulation of the extremity joints. The length of time that these changes last, however, is unclear beyond an immediate effect.

## Data Availability

The datasets used and/or analyzed during the current study are available from the corresponding author on reasonable request.

## Abbreviations

IMU: Inertial Measurement Unit
SD: Standard Deviation
ML: Medial to lateral
SampEn: Sample Entropy
COP: Center of Pressure
Pathlength: Path Length
RMS: Root Mean Square ANOVA Analysis of Variance
